# Changes in metamorphopsia following intravitreal aflibercept injection for diabetic macular edema

**DOI:** 10.1038/s41598-022-22401-y

**Published:** 2022-10-17

**Authors:** Tomoya Murakami, Fumiki Okamoto, Yoshimi Sugiura, Shohei Morikawa, Yoshifumi Okamoto, Takahiro Hiraoka, Tetsuro Oshika

**Affiliations:** 1 Department of Ophthalmology, Faculty of Medicine, University of Tsukuba, 1-1-1 Tennoudai, Tsukuba, Ibaraki 305-8575 Japan; 2 Mito Kyodo General Hospital, Ibaraki, Japan

**Keywords:** Eye diseases, Retinal diseases

## Abstract

The aim of the present study was to investigate changes in metamorphopsia in patients with diabetic macular edema (DME) following intravitreal aflibercept injection (IVA) with the treat and extend (TAE) regimen for a year. We performed a post hoc analysis of a multicenter, open-label, single-arm, prospective study. The study included 20 patients with DME. All eyes received 3 monthly loading injections of 2 mg aflibercept, followed by a TAE regimen. Every visit, the severity of metamorphopsia and the best-corrected visual acuity (BCVA) were evaluated, and optical coherence tomography (OCT) images were obtained. The severity of metamorphopsia was measured using M-CHARTS. The metamorphopsia scores before treatment and at 1, 2, 3, 6 and 12 months following treatment were 0.25 ± 0.23, 0.21 ± 0.15, 0.19 ± 0.23, 0.14 ± 0.16, 0.17 ± 0.20 and 0.10 ± 0.17, respectively, with significant improvement from before treatment to 3 and 12 months following treatment (p < 0.05 and p < 0.005, respectively). At the time of macular edema resolution, the presence of an epiretinal membrane (ERM) was associated with the metamorphopsia score (p < 0.05). In conclusion, the metamorphopsia score in patients with DME improved following IVA with the TAE regimen for one year. The presence of ERM was associated with the metamorphopsia score.

## Introduction

Diabetic macular edema (DME) is one of the most important vision-threatening conditions in patients with diabetic retinopathy^1^. The visual prognosis of patients with DME has improved after the introduction of anti-vascular endothelial growth factor (VEGF) therapy^2–5^. However, in a clinical setting, some patients with DME experience symptomatic metamorphopsia.

Metamorphopsia is a common symptom in patients with retinal disorders, such as epiretinal membrane (ERM)^6,7^, macular hole (MH)^8,9^, retinal detachment (RD)^10,11^, and macular edema secondary to retinal vein occlusion^12–16^. Several researchers have investigated the relationship between metamorphopsia and vision-related quality of life (VR-QoL) and reported that metamorphopsia was more strongly associated with VR-QoL than visual acuity in patients with ERM^7^ and MH^9^ and who underwent RD surgery^11^. Although metamorphopsia may be important in retinal disorders, little information has been obtained on metamorphopsia in patients with DME.

Nowacka et al.^17^ investigated the incidence of metamorphopsia in patients with DME undergoing anti-VEGF therapy (3 monthly intravitreal injections of ranibizumab (IVR) followed by a pro re nata (PRN) approach). They reported that after 3 months, the percentage of patients complaining of metamorphopsia significantly decreased from baseline; however, this number increased after 6 months, with no significant difference compared to before treatment. Okamoto et al.^18^ investigated metamorphopsia in patients undergoing intravitreal injection of bevacizumab for persistent DME after vitrectomy and reported that the severity of metamorphopsia was not improved 3 months after treatment. However, no previous reports have described the long-term quantitative evaluation of metamorphopsia in patients with DME.

It has been reported that aflibercept is more effective than ranibizumab in some subgroups of patients with DME^19^. The treat-and-extend (TAE) regimen is the next most commonly used treatment regimen for DME after the PRN regimen^20^. Moreover, the TAE regimen has the advantage of reducing the number of hospital visits^21^, and its use is expected to increase during the COVID-19 pandemic. However, to the best of our knowledge, there is no information available on metamorphopsia in patients with DME treated with aflibercept using the TAE regimen. Therefore, the purpose of the current study was to investigate metamorphopsia in DME eyes treated with aflibercept using a TAE regimen for one year.

## Results

We included 20 eyes of 20 patients with DME. Table 1 lists the baseline clinical characteristics. The mean number of injections was 6.7 ± 1.2. No significant adverse events, including endophthalmitis, RD, or cataract development, were observed during the study period. Throughout the follow-up period, none of the patients received cataract surgery.

**Table 1:** Baseline clinical characteristics.

No. of eyes	20
Age, years	61.8 ± 10.7
Sex (male/female)	7/13
Metamorphopsia score	0.25 ± 0.23
BCVA (logMAR)	0.41 ± 0.34
CFT (µm)	468 ± 227

*BCVA* best-corrected visual acuity, *logMAR* logarithm of the minimum angle of resolution, *CFT* central foveal thickness.

### Changes in visual functions

**Figure 1:**
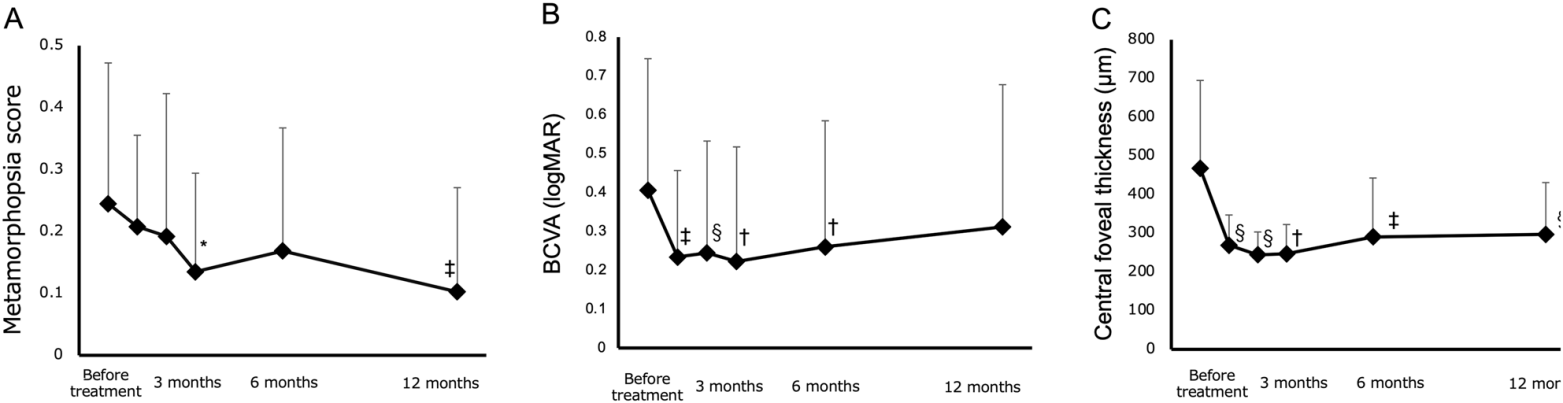
Changes in metamorphopsia score (**A**), best correlated visual acuity (BCVA) (**B**) and central foveal thickness (CFT) (**C**) from baseline in eyes treated with intravitreal aflibercept injection (IVA) for diabetic macular edema (DME). Error bars indicate standard deviations. Statistically significant compared to baseline (*p < 0.05, ^†^p < 0.01, ^‡^p < 0.005, ^§^p < 0.001).

Significant metamorphopsia (score ≥ 0.2) was detected in 10 of 20 eyes before treatment and in 4 of 20 eyes 12 months after treatment. The percentage of patients with significant metamorphopsia decreased significantly from before treatment to 12 months after treatment (p < 0.05). The changes in visual functions and the central foveal thickness (CFT) are shown in Fig. 1. The metamorphopsia scores before treatment and at 1, 2, 3, 6 and 12 months following treatment were 0.25 ± 0.23, 0.21 ± 0.15, 0.19 ± 0.23, 0.14 ± 0.16, 0.17 ± 0.20 and 0.10 ± 0.17, respectively. Three and twelve months after treatment, metamorphopsia scores were significantly improved compared to those bofore treatment. (p < 0.05 and p < 0.005, respectively) (Fig. 1A). The best-corrected visual acuity (BCVA) before treatment and at 1, 2, 3, 6 and 12 months following treatment were 0.41 ± 0.34, 0.23 ± 0.22, 0.24 ± 0.29, 0.22 ± 0.29, 0.26 ± 0.32 and 0.31 ± 0.37, respectively. BCVA was significantly improved at 1, 2, 3, and 6 months following treatment compared to baseline (p < 0.005, p < 0.001, p < 0.01 and p < 0.01, respectively), although no significant difference was observed between 12 months after treatment and before treatment (Fig. 1B). The CFT values before treatment and at 1, 2, 3, 6 and 12 months following treatment were 468 ± 227, 269 ± 78, 244 ± 59, 247 ± 76, 291 ± 152 and 290 ± 134, respectively. CFT was significantly improved at 1, 2, 3, 6 and 12 months following treatment comparerd to baseline (p < 0.001, p < 0.001, p < 0.01, p < 0.005 and p < 0.001, respectively) (Fig. 1C).

**Figure 2:**
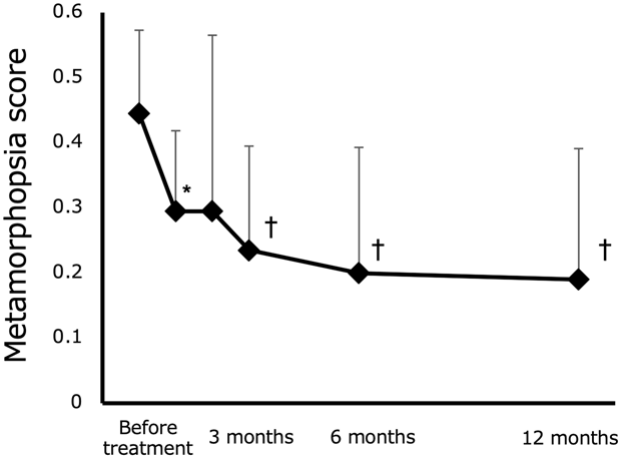
Changes in metamorphopsia scores of patients with diabetic macular edema (DME) with metamorphopsia at baseline. Error bars indicate standard deviations. Statistically significant compared to baseline (*p < 0.05, ^†^p < 0.01).

A subgroup analysis of ten patients who had significant metamorphopsia (score ≥ 0.2) before treatment revealed that the mean metamorphopsia scores before treatment and at 1, 2, 3, 6 and 12 months following treatment were 0.45 ± 0.13, 0.30 ± 0.12, 0.30 ± 0.27, 0.24 ± 0.16, 0.20 ± 0.19 and 0.19 ± 0.20, respectively. Metamorphopsia scores were significantly improved at 1, 3, 6, and 12 months following treatment compared to those before treatment. (p < 0.05, p < 0.01, p < 0.01 and p < 0.01, respectively) (Fig. 2). No patient without metamorphopsia before treatment developed metamorphopsia 12 months following treatment.

**Figure 3:**
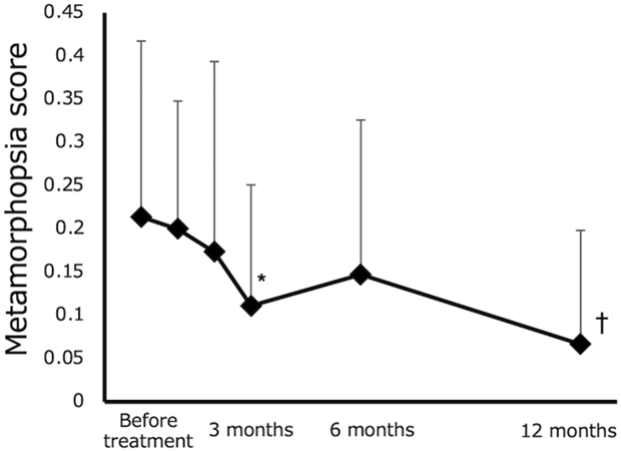
Changes in metamorphopsia scores of patients without epiretinal membrane. Error bars indicate standard deviations. Statistically significant compared to baseline (*p < 0.01, ^†^p < 0.005).

ERM was observed in two patients. A subgroup analysis of 18 patients without ERM revealed that the mean metamorphopsia scores before treatment and at 1, 2, 3, 6 and 12 months following treatment were 0.23 ± 0.20, 0.20 ± 0.15, 0.17 ± 0.23, 0.11 ± 0.14, 0.13 ± 0.16 and 0.07 ± 0.13, respectively. Metamorphopsia scores were significantly better at 3 and 12 months following treatment than those before treatment. (p < 0.01, p < 0.005, respectively) (Fig. 3).

### Relationship between metamorphopsia and visual acuity

The metamorphopsia score before treatment was not significantly correlated with BCVA before treatment (r = 0.031, p = 0.896). The metamorphopsia score 12 months following treatment was also not significantly correlated with BCVA (r = 0.085, P = 0.721).

### Relationship between visual functions and OCT findings

The association between visual functions and OCT findings before treatment is shown in Table 2. OCT parameters were not significantly associated with the mean metamorphopsia score. The BCVA of eyes with an external limiting membrane (ELM) grade 0, grade 1, and grade 2 was 0.21 ± 0.18, 0.42 ± 0.26 and 1.13 ± 0.04, respectively, and ELM status was significantly associated with BCVA (p = 0.018). The BCVA of eyes with an ellipsoid zone (EZ) grade 0, grade 1, and grade 2 was 0.10 ± 0.00, 0.33 ± 0.24 and 0.81 ± 0.40, respectively, and EZ status was significantly associated with BCVA (p = 0.042). Other OCT findings were not associated with BCVA.

**Table 2:** Correlations between visual functions and OCT findings before treatment.

Factors	Metamorphopsia score (*p* value)	BCVA (logMAR) (*p* value)
ELM	0.175	0.018*
EZ	0.590	0.042*
Inner cyst	0.683	0.392
Outer cyst	0.300	0.250
SRD	0.224	0.388
ERM	0.050	0.250
CFT	0.806 (R = 0.059)	0.208 (R = 0.294)

*BCVA* best-corrected visual acuity, *ELM* external limiting membrane, *EZ* ellipsoid zone, *SRD* serous retinal detachment, *ERM* epiretinal membrane, *CFT* central foveal thickness.

*Significant correlations found between the parameters (Kruskal–Wallis test, *p < 0.05).

The association between visual functions and OCT findings when macular edema was resolved is shown in Table 3. In 13 eyes, DME was most resolved 12 months following treatment. In four eyes, DME was most resolved 9 months following treatment. In three eyes, DME was most resolved 6 monthes following treatment. ERM was observed in two patients. The mean metamorphopsia of eyes with ERM and without ERM were 0.35 ± 0.35 and 0.05 ± 0.11, respectively, and the presence of ERM was significantly correlated with the mean metamorphopsia score (p = 0.036), whereas other parameters were not correlated.

**Table 3:** Correlations between visual functions and OCT findings at the time the macular edema was mostly resolved.

Factors	Metamorphopsia score (*p* value)	BCVA (logMAR) (*p* value)
ELM	0.274	0.446
EZ	0.447	0.523
Inner cyst	0.651	0.837
Outer cyst	1.000	0.962
ERM	0.036*	0.277
CFT	0.251 (R = −0.285)	0.405 (R = 0.203)

*BCVA* best-corrected visual acuity, *ELM* external limiting membrane, *EZ* ellipsoid zone, *SRD* serous retinal detachment, *ERM* epiretinal membrane, *CFT* central foveal thickness.

*Significant correlations found between the parameters (Mann–Whitney U test, *p < 0.05).

## Discussion

The metamorphopsia score in DME patients was significantly improved from baseline to 3 and 12 months after treatment for TAE regimen in this study. Nowacka et al.^17^ investigated the incidence of metamorphopsia in patients with DME undergoing anti-VEGF therapy (3 monthly intravitreal injections of ranibizumab (IVR) followed by PRN). They reported that 88.2% of the patients had metamorphopsia before treatment, which significantly decreased to 41.2% at 3 months after treatment; however, 70.6% had metamorphopsia at 6 months after treatment, with no significant difference from before treatment. Based on the results of this study and a previous study, metamorphopsia is expected to improve after 3 loading injections at the beginning of the treatment. In the previous study, the percentage of patients with metamorphopsia did not significantly decrease from baseline to 6 months following treatment. In contrast, the metamorphopsia score was significantly improved from baseline to 12 months following treatment in this study. The cause of the discrepancy between the findings of this study and those of the previous study is unclear; however, the type of drugs used and the treatment regimen may have affected the results. The Diabetic Retinopathy Clinical Research Network reported that in patients with DME whose BCVA is 20/50 or worse, aflibercept is more effective at improving vision than ranibizumab^19^. Moreover, it was also reported that conversion to aflibercept for DME with a suboptimal response to ranibizumab resulted in significant anatomic improvements^22^ or significant BCVA improvements^23^. Based on these previous reports, we considered that aflibercept might be superior to ranibizumab for DME treatment. However, more studies are needed to prove whether aflibercept is superior to ranibizumab in reducing the severity of the metamorphopsia in patients with DME.

Because this was a single-arm study, we could not evaluate whether the treatment approach such as TAE or PRN would involve the long-term effect on metamorphopsia. No researchers have compared the prognosis of metamorphopsia among eyes with DME treated with TAE regimen and those treated with PRN regimens. Lai et al. compared the 2-year treatment outcomes of ranibizumab using TAE or PRN regimens and reported that the TAE group had more visual acuity gain and anatomical improvement than the PRN group^24^. Several researchers have investigated changes in metamorphopsia after anti-VEGF therapy for macular edema (ME) secondary to branch retinal vein occlusion (BRVO)^13–15^. A study of patients treated with the TAE regimen showed that metamorphopsia improved significantly from baseline 12 months after treatment^15^, while patients treated with the PRN regimen showed no metamorphopsia improvement^13,14^. Based on the results of this study and those of previous studies, metamorphopsia is more likely to improve after treatment with the TAE regimen than with the PRN regimen. However, more studies are needed to prove whether TAE regimen is superior to PRN regimen in reducing severity of the metamorphopsia in patients with DME. Mori et al.^15^ investigated the changes in metamorphopsia after intravitreal injections of anti-VEGF agents (ranibizumab or aflibercept) using a TAE regimen for ME secondary to BRVO. They reported that the metamorphopsia score was significantly improved after 12 months of treatment (median value, 0.4) from baseline (median value, 0.45)^15^. In this study, in the ten cases in which metamorphopsia was detected at baseline, the metamorphopsia score improved from 0.45 ± 0.13 at baseline to 0.19 ± 0.20 12 months following treatment. The changes in the metamorphopsia score in our study were more remarkable than those in previous studies on ME secondary to BRVO. The discrepancy between metamorphopsia prognosis in patients with ME secondary to BRVO and those with DME is unknown. DME is typically chronic, while ME secondary to BRVO is acute. We speculate that this difference might be responsible for the discrepancy.

In this study, when DME was mostly resolved, the presence of ERM was significantly associated with the metamorphopsia score. It has been reported that ERM is present in 6.2% of DME eyes^25^, and ERM was found in 9.5% of patients with DME during 2 years of anti-VEGF treatment^26^. In this study, one patient (5%) had ERM at baseline, and one patient (5%) developed ERM during the observation period, which is consistent with the findings of previous studies^25,26^. It has been reported that metamorphopsia improves in ERM eyes after vitrectomy^7,27^; thus, we speculate that patients with DME complicated by ERM whose quality of vision is impaired by metamorphopsia may benefit from vitrectomy. Baseline ELM and EZ were significantly associated with BCVA in this study, and these findings were consistent with previous studies^28,29^.

The limitations of this study include the relatively small number of participants. Because this was a single-arm study, it is not clear whether the TAE regimen is superior to the PRN regimen or whether aflibercept is superior to ranibizumab for improving metamorphopsia in patients with DME. Furthermore, this was a post hoc analysis. Future two-arm studies with a larger sample size are warranted to identify drugs or treatment regimens that are highly effective in improving metamorphopsia.

In conclusion, the proportion of DME patients with metamorphopsia significantly decreased after 1 year of treatment with aflibercept using the TAE regimen. The metamorphopsia score also significantly improved after 1 year of treatment. With the resolution of macular edema, the presence of ERM was significantly associated with the metamorphopsia score.

## Methods

We performed a post hoc analysis of a multicenter, open-label, single-arm, prospective study (UMIN ID: UMIN000020324). The study protocol of original study was approved by the Institutional Review Board of Tsukuba University Hospital and Mito Kyodo General Hospital (approval number: H27-237), and the study was conducted in accordance with the tenets of the Declaration of Helsinki. All patients provided their written informed consent prior to study participation. The original study enrolled patients with DME who were referred to the Tsukuba University Hospital or Mito Kyodo General Hospital between July 2015 and December 2018. Patients were eligible for enrollment if they met all of the following criteria: (1) age > 20 years; (2) diagnosis of center-involving DME (central retinal thickness ≥ 300 μm on optical coherence tomography); and (3) BCVA of 20/320 or better (Snellen visual acuity). The exclusion criteria were as follows: (1) previous history of ophthalmic disorders in both eyes, except mild refractive errors, mild cataract, and stage 1 ERM^30^ located outside of fovea; (2) history of vitreoretinal or glaucoma surgery in the study eye; (3) anti-VEGF treatment and laser retinal photocoagulation in the study eye within 90 days prior to enrollment; (4) intra/peri-ocular steroid injection in the study eye within 120 days prior to enrollment; and (5) severe diabetes (hemoglobin A1c > 12%), hypertension (systolic blood pressure > 160 mmHg and diastolic blood pressure > 95 mmHg) and renal failure. In patients with DME in both eyes, we enrolled the eye with worse visual acuity as the study eye. Twenty patients for whom metamorphopsia data were available were enrolled in this post hoc analysis.

### Assessments

We examined the severity of metamorphopsia using M-CHARTS (Inami Co) and BCVA at each visit. The principle behind the M-CHARTS has been described in detail previously^7,9–16^. Briefly, the M-CHARTS comprises 19 dotted lines with dot intervals ranging from 0.2° to 2.0° of visual angle. The patients viewed the M-CHARTS from a distance of 30 cm with appropriate correction. When the straight line is substituted with a dotted line and the dot interval is changed from fine to coarse, the line distortion reduces with the enhanced dot interval until the line seems straight. First, we showed a patient vertical straight line (0°). If the patient recognized a straight line as straight, the metamorphopsia score was 0. If the patient recognized a straight line as irregular or curved, we showed subsequent pages of M-CHARTS, where the dot intervals of the dotted line changed from fine to coarse, one after another. When the patient recognized a dotted line as straight, the visual angle separating the dots was determined to represent their metamorphopsia score for vertical lines. Then, the M-CHARTS was rotated 90°, and the same test was performed with horizontal lines. The average metamorphopsia score of vertical and horizontal metamorphopsia scores was used for the analysis. The examinations were repeated three times, and the mean values were used for data analyses.

Spectral-domain optical coherence tomography (Cirrus high-definition OCT; Carl Zeiss, Dublin, CA) images were recorded at each visit. We performed Macular cube 512 × 128 scans and five-line raster cross scans using the Cirrus analysis software V.3.0. Scans with a signal strength equal to or higher than 7/10 wereconsidered to be appropriate. OCT findings, including the status of the ELM and EZ, the presence of inner and outer retinal cysts, serous retinal detachment (SRD), and the ERM and CFT were obtained based on the OCT images. The ELM and EZ status was classified into three grades: grade 0: continuous, grade 1: disrupted, and grade 2: absent. We evaluated the status of the ELM and EZ and the presence of retinal cysts, SRD and ERM at a 3 mm area centered on the fovea in the vertical OCT images. We measured the CFT manually using Cirrus analysis software. The OCT findings were assessed by two evaluators (TM and YS). Both graders were masked to the clinical findings of the patients. When graders’ evaluations differed, a consensus was reached by consulting one of the authors (FO), who was blind to the clinical results.

### Treatment

The eyes received three consecutive intravitreal injections of 2 mg aflibercept every four weeks. The injection intervals were then adjusted by four weeks based on OCT findings. If the CFT increased more than 100 μm above the lowest previously measured value, the CFT was 350 µm or more, or a new intraretinal cyst or SRD was detected, the interval was shortened (minimum 4 weeks). The interval was extended if these findings were not observed (maximum 16 weeks).

### Statistical analyses

The BCVA was measured with a Landolt chart and was converted to the logarithm of the minimum angle of resolution (logMAR) for statistical analyses. The Wilcoxon signed-rank test was used to assess changes in visual functions (metamorphopsia score and BCVA) and CFT. Fisher’s exact test was used to evaluate changes in the percentage of patients with significant metamorphopsia (metamorphopsia score ≥ 0.2). We investigated the relationship between visual functions and OCT findings at baseline and at the time of the final visit when macular edema had resolved. We used the Kruskal–Wallis test to compare visual functions between the three groups according to the status of the ELM and EZ. We used the Mann–Whitney U test to compare the visual functions between the two groups according to the presence of outer retinal cysts, inner retinal cysts, SRD and ERM. We examined the correlation between visual functions and CFT using the Spearman rank correlation coefficient.

## Data Availability

The data that support the findings of this study are available from the corresponding author, T.M., upon reasonable request.
